# Results of open bleb revision as management of primary bleb failure following XEN 45 gel stent and Preserflo™ Microshunt

**DOI:** 10.1007/s00417-023-06152-8

**Published:** 2023-07-06

**Authors:** Theresa Theilig, Menelaos Papadimitriou, Ghaith Albaba, Daniel Meller, Somar M. Hasan

**Affiliations:** Klinik für Augenheilkunde, Universitätsklinikum Jena, Am Klinikum 1, 07747 Jena, Germany

**Keywords:** Bleb revision, XEN, Preserflo, Filtering glaucoma surgery, MIGS

## Abstract

**Purpose:**

The success of filtering surgery as in XEN-Gel-Stent (XEN) and Preserflo-Microshunt (PF) depends mainly on a functioning bleb. Primary bleb failure (PBF) is not uncommon and can be treated with needling or open bleb revision (OBR). The aim of the study is to compare surgical outcomes of OBR after XEN and PF.

**Methods:**

Eyes which received OBR as management of PBF following implantation of XEN or PF were retrospectively included. Intraocular pressure (IOP), number of IOP lowering medications (NoM), and success rates (SR) were compared between groups. Complete and qualified success were defined as IOP ≤18mmHg and a reduction of >20%, without and with medications, respectively.

**Results:**

29 eyes after XEN and 23 eyes after PF were included. Six months following OBR, IOP reduced from 24.2±4.7 to 13.5±4.6 after XEN and from 27.3±8.7 to 15.9±5.8mmHg after PF (both *p*<0.001). NoM did not change (0.7±1.3 to 0.4±0.8 after XEN and 1.2±1.3 to 1.0±1.5 after PF, *p*>0.05 for both). Complete SR were higher after XEN than after PF (58.6% vs. 30.4%, *p*=0.04). Complications were mild and managed mainly conservatively. Additional glaucoma surgery was needed in 17% and 30% of eyes after XEN and PF, respectively (*p*=0.26).

**Conclusion:**

Although OBR was effective as management of PBF following XEN and PF, SR were higher after XEN than after PF along with comparable safety profile. The change of the surgical approach from ab interno during XEN-Implantation to ab externo during OBR seems to enhance SR compared to PF, where both interventions are done ab externo.

## Introduction


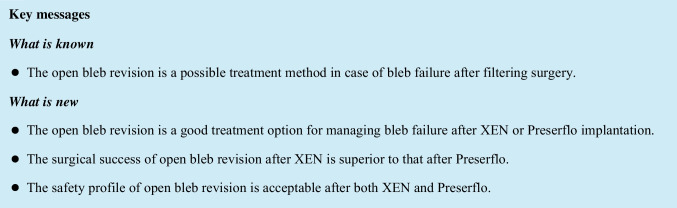


Trabeculectomy (TE) has been the gold standard of glaucoma surgery for many years [1]. Minimally invasive glaucoma surgery (MIGS), specifically minimally invasive bleb surgery (MIBS), is aimed at providing alternative options to TE with similar efficacy and improved safety. These alternatives base on the implantation of stents and are aimed at draining aqueous humor from the anterior chamber into the subconjunctival space [2]. Examples include XEN45-Gel-Stent (XEN, Allergan Inc., CA, USA, an Abbvie company, IL, USA) (XEN) and Preserflo™ MicroShunt (Santen Pharmaceutical Co., Osaka, Japan) (PF) which use different surgical approaches (ab interno vs. ab externo, respectively). Both procedures effectively reduce intraocular pressure (IOP) even compared to the TE with tolerable safety profile [3, 4], but can still result in primary bleb failure (PBF), occurring in up to 62% of XEN [5] and up to 19% of PF cases [4]. PBF with IOP exceeding target pressure mostly demands surgical management either with needling or with open bleb revision (OBR). Although OBR seems to deliver better results compared with needling after XEN [6], the current data regarding the results of OBR after PF are very scarce. However, these data are essential for the decision-making process in case of PBF. Applying the same surgical method (OBR) following two different procedures (XEN vs. PF) may not imply comparable outcomes. Our study is aimed at reporting the outcomes of OBR for PBF after XEN and PF and comparing their results.

## Materials and methods

This retrospective study included eyes that underwent OBR for PBF after XEN or PF implantation at the Department of Ophthalmology, Jena University Hospital in Germany. Included were patients diagnosed with primary open angle glaucoma (POAG) and pseudoexfoliation glaucoma (PEXG) and completed a minimum of 6 months of follow-up. The indication for OBR was made in patients showing PBF diagnosed morphologically while exceeding the target pressure set by the glaucoma specialist.

Preoperative demographic data including age, gender, laterality, lens status, ocular surgical history, and usage of antimetabolites were noted. Measurements taken preoperatively (before primary implantation of XEN or PF and again before OBR) and postoperatively following OBR (at discharge and 1, 3, and 6 months) included IOP measured by Goldmann applanation tonometry, the number of IOP lowering medication (NoM), mean defect (MD) of static automated perimetry testing (Octopus 900 EyeSuite program: 30°-2, Haag-Streit International, Köniz, Switzerland), and intra- and post-operative complications and re-operations. Surgical success was defined as follows: complete success (CS) was achieved if IOP was ≤18 mmHg and >5mmHg without IOP lowering medications with an IOP reduction of ≥20% compared to preoperative IOP (before OBR). Qualified success (QS) was achieved at the same IOP reduction and level as in CS but with IOP lowering medications (not exceeding the NoM before OBR). Failure included further glaucoma surgery or loss of light perception.

The study followed the guidelines of the Declaration of Helsinki. Statistical analysis was performed using SPSS (SPSS Statistics 27.0, IBM Corporation, Armonk, NY, USA). Success rates (SR) and patients’ characteristics were assessed using a chi-square test. IOP, NoM, and MD were assessed using dependent and independent *t*-test within and between the XEN and PF groups, with *p*<0.05 indicating statistical significance.

### Surgical technique

In the primary surgery, XEN implantation was performed through an ab interno-approach with subconjunctival injection of mitomycin C (MMC) (0.2mg/ml, 0.1ml) without primary needling. PF was implanted through an ab externo approach with application of MMC (0.2mg/ml) for 2 minutes.

During the OBR, a fornix-based conjunctival opening was performed over 2 clock hours in the bleb area, the sclera was exposed, and tenon scar tissue around the implant was dissected bluntly from the periphery towards the implant and then removed, taking care not to damage the implant. The drainage through the implant was checked and 2 corneal sponge shields (8mm corneal light shields, BVI Visitec®, Beaver-Visitec International, Inc., MA, USA) soaked with MMC (0.2mg/ml) were applied in the episcleral space for 2-3 minutes and then removed. The MMC was extensively rinsed out with balanced salt solution. Tenon was then sutured to the sclera using 2 interrupted absorbable sutures (10-0 Vicryl, Ethicon, Somerville, NJ, USA) ensuring that the outer segment of the implant is located in the subtenon space. This was followed by watertight closure of the conjunctiva (10-0 Vicryl). In those cases where no MMC was used, the patients received 5-fluorouracil (5-FU) (10mg/ml, 0.5ml) postoperatively as subconjunctival injection once a day. Treatment was repeated as decided by glaucoma specialist.

Postoperatively, the patients received ofloxacin eye drops 5 times a day for 1 month (Floxal® 3mg/ml eye drops, Bausch&Lomb, Laval, Canada) and dexamethasone eye drops (Dexapos COMOD, URSAPHARM, Saarbrücken, Germany) q2h for 1 week, then 5 times a day with monthly reduction of 1 drop.

## Results

Included were 52 eyes from 51 patients, 29 eyes after XEN revision (XENR), and 23 eyes after Preserflo revision (PFR). The demographic data and patients’ characteristics can be found in Table 1. There were no significant differences between the two groups regarding age, sex, laterality, diagnosis, lens status (phakic versus pseudophakic), preoperative IOP, NoM, time point between primary surgery, and OBR or the used antimetabolite. Prior glaucoma surgery was slightly less common in the XENR group with 2x selective laser trabeculoplasty and 1x cyclophotocoagulation (CPC) versus 2xCPC and 2xTE in the PFR group, without significant difference (*p*=0.46). The MD value of perimetry was higher in PFR compared to XENR (11.2±6.9 vs. 5.7±4.3, *p*=0.002). If MMC was used, the application time of MMC was significantly lower in XENR than in PFR (2.1±0.4 vs. 2.6±0.8 minutes, *p*=0.028).

**Table 1:** Demographic data and patients’ characteristics, specification as mean ± standard deviation (*m*) or number in absolute and relative terms (*n*)

			XENR (*n*=29)		PFR (*n*=23)		*p*-value
Age (years)		*m*	70.5	±6.6	73.3	±6.9	0.144^+^
Gender	Males	*n*	9	(31%)	5	(22%)	0.453^‡^
	Females		20	(69%)	18	(78%)	
Laterality	Right	*n*	12	(41%)	10	(43%)	0.879^‡^
	Left		17	(59%)	13	(57%)	
Glaucoma diagnosis	POWG	*n*	24	(83%)	18	(78%)	0.683^‡^
	PEXG		5	(17%)	5	(22%)	
Prior glaucoma surgery	No	*n*	26	(90%)	19	(83%)	0.460^‡^
	Yes		3	(10%)	4	(17%)	
Pseudophakia	No	*n*	18	(62%)	10	(43%)	0.168^‡^
	Yes		11	(38%)	13	(57%)	
Used antimetabolite	MMC	*n*	16	(55%)	20	(86%)	0.158^‡^
	5-FU		10	(34%)	3	(14%)	
	Combination None		2 1	(7%) (3%)	0 0	(0%) (0%)	
MMC application time (minutes)		*m*	2.1	±0.4	2.6	±0.8	0.028^+^
IOP (before OBR) (mmHg)		*m*	24.2	±4.7	27.3	±8.7	0.178^+^
NoM (before OBR)		*m*	0.7	±1.3	1.2	±1.3	0.178^+^
MD (before OBR) (dB)		*m*	5.7	±4.3	11.2	±6.9	0.002^+^
Time period from primary surgery until OBR (days)		*m*	170.3	±265.6	129.1	±112.1	0.490^+^

^+^Independent *t*-test

^‡^Chi-square test

Postoperatively, IOP, NoM, and MD were considered only of eyes without further glaucoma surgery (following OBR) at the relevant time point.

**Fig. 1:**
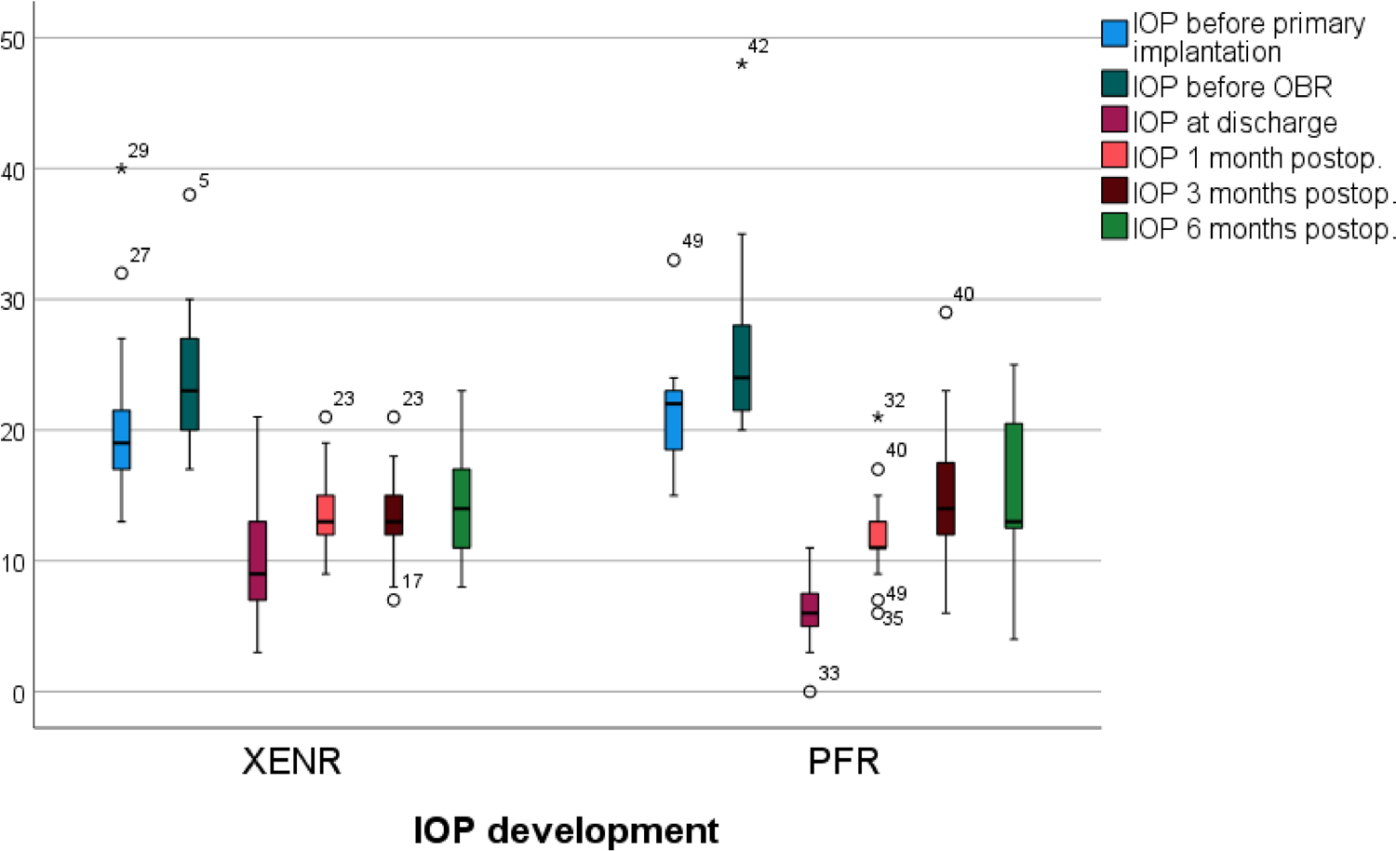
IOP development before primary surgery, at the indication of OBR and postoperatively at discharge, and after 1, 3, and 6 months. Data in mmHg

**Fig. 2:**
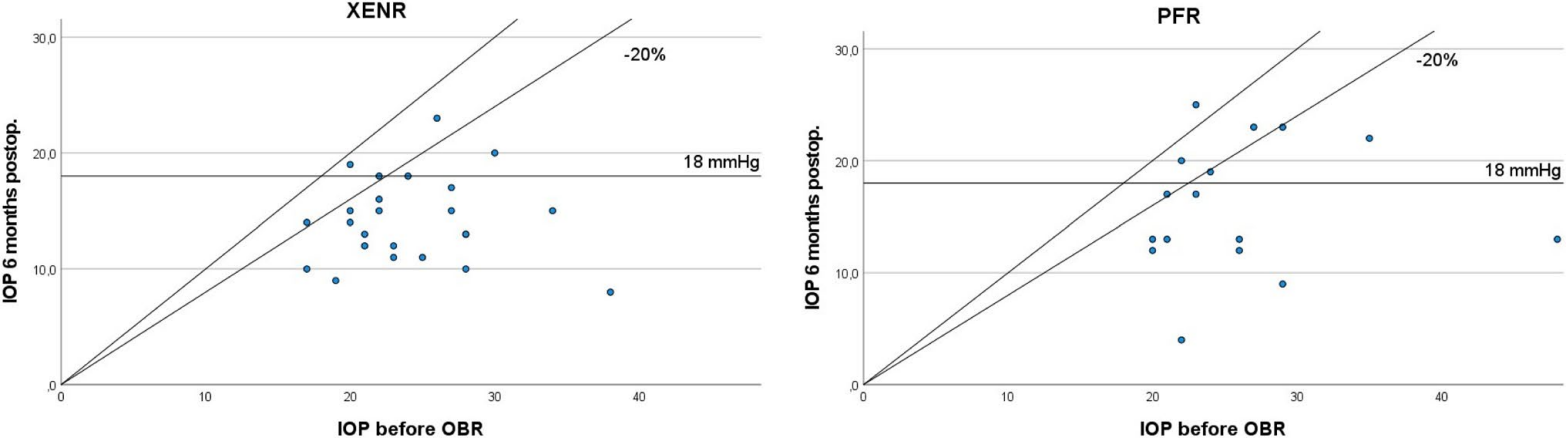
Scatterplot comparing preoperative to 6 months postoperative IOP in the XENR and PFR groups. Data in mmHg

Postoperative IOP levels (listed in Table 2) significantly decreased in both groups at all time points (at discharge, 1, 3, and 6 months) compared to pre-OBR levels (*p*<0.001 for all, Figs. 1 and 2). Intergroup comparison showed a higher IOP in the XENR group at discharge compared to the PFR (*p*=0.001), but no significant difference was found at later visits (1, 3, and 6 months, *p*>0.05 for all).

**Table 2:** IOP and NoM before primary surgery, before OBR, and postoperatively at discharge and after 1, 3, and 6 months

		XENR			PFR			
	Visit	Mean	±SD	*p*-value^+^	Mean	±SD	*p*-value^+^	*p*-value^‡^
IOP (mmHg)	Before primary surgery	21.9	±8.2		24.1	±6.6		0.307
	Before OBR	24.2	±4.7		27.3	±8.7		0.109
	Discharge	11.4	±5.4	<0.001	6.9	±3.7	<0.001	0.001
	1 month	14.3	±3.2	<0.001	14.5	±6.0	<0.001	0.896
	3 months	14.0	±3.9	<0.001	18.6	±11.3	<0.001	0.082
	6 months	13.5	±4.6	<0.001	15.9	±5.8	<0.001	0.140
NoM	Before primary surgery	2.6	±1.5		3.4	±1.2		0.045
	Before OBR	0.7	±1.3		1.2	±1.3		0.178
	Discharge	0.4	±1.1	0.067	0.1	±0.4	<0.001	0.227
	1 month	0.4	±0.9	0.188	0.2	±0.7	0.001	0.295
	3 months	0.5	±0.9	0.377	0.9	±1.5	0.438	0.227
	6 months	0.4	±0.8	0.641	1.0	±1.5	0.362	0.097

*SD* standard deviation

^+^Dependent *t*-test related to values before OBR

^‡^Independent *t*-test between XENR and PFR

The NoM did not change following OBR in the XENR (*p*>0.05 for all). A reduction of NoM till 1 month in the PFR (*p*=0.001 at 1 month) was observed. However, on the 3- and 6-month visits, this difference also disappeared (*p*>0.05 for all). Intergroup comparison showed no difference in the NoM at any time point.

Within the first 6 months, MD of the perimetry improved respectively remained stable in both groups (*p*=0.031 XENR, *p*=0.083 PFR).

### Success rates

Within the 1- and 3-month visits, CS and QS rates did not differ significantly between XENR and PFR (*p*=0.51 resp. *p*=0.57). Six months after OBR SR were significantly higher after XENR at 58.6% compared to 30.4% after PFR (*p*=0.043). There were no differences between CS and QS in both groups (Table 3).

**Table 3:** Success rates at 6 months postoperatively after OBR

	Complete success		Qualified success		Failure		*p*-value^+^
	XENR	PFR	XENR	PFR	XENR	PFR	
1 month	60.7%	69.6%	64.3%	69.6%	35.7%	30.4%	0.510
3 months	56.0%	47.8%	56.0%	47.8%	44.0%	52.2%	0.571
6 months	58.6%	30.4%	58.6%	30.4%	41.8%	69.6%	0.043

^+^Chi-square test

**Fig. 3:**
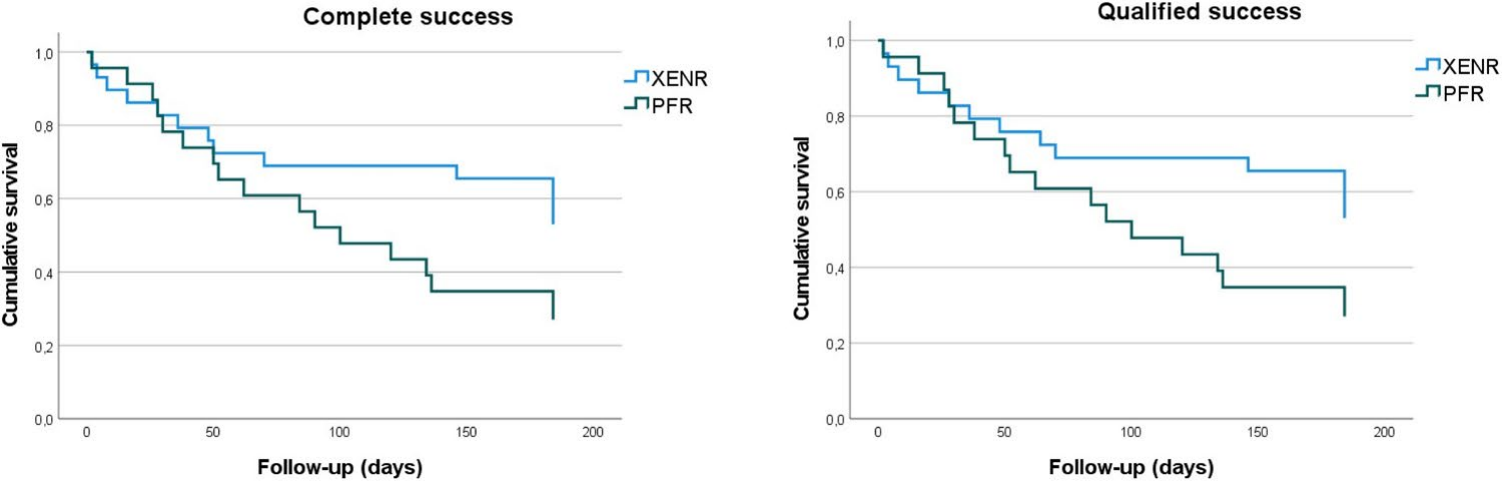
Cumulative survival of CS and QS after OBR; *p*=0.065 resp. *p*=0.060 (log rank test)

Figure 3 shows the Kaplan-Meier-curves of CS and QS rates after XENR and PFR. Log rank test approaches significance with *p*=0.065 for CS and *p*=0.060 for QS.

Almost half of the failures in both groups can be traced back to repeated glaucoma surgery. With XENR, the rate of re-surgery was 17% after 6 months (4xTE, 1xPF). After PFR it was slightly higher with 30% (3xCPC, 1xCPC following PF explantation, 2xOBR, and 1x Paul implant).

Complications, listed in Table 4, were infrequent. Further non-IOP-lowering operations were performed more frequently in eyes after XENR: 3x conjunctival sutures in case of 2x dehiscence and 1x positive Seidel test, 1x anterior chamber irrigation, compared to PFR with 1x anterior vitrectomy because of vitreous prolapse with occlusion of the implant, 1x ischemic conjunctiva with a button hole defect treated by conjunctival resection followed by explantation of the implant because of implant exposure. A positive Seidel test was managed conservatively in one case of each group; in another case, surgical intervention was needed in each group (see above). There were no cases of persistent hypotony over >30 days, hypotonic maculopathy, endophthalmitis, aqueous misdirection, or loss of light perception.

**Table 4:** Postoperative complications and re-operations

	XENR (*n*=29)	PFR (*n*=23)
Choroidal detachment	3	3
Conjunctival dehiscence	3	0
Exposure of the implant (following surgery because of ischemic conjunctiva)	0	1
Vitreous prolapse	0	1
Positive Seidel test	2	1
Non-IOP-lowering re-operations	4	2
IOP-lowering re-operations	5	7

## Discussion

Minimal invasive bleb surgeries such as XEN and PF are commonly used modern procedures, known for their effective pressure and drug reduction with low risks of complications [3, 4, 7, 8]. Nevertheless, PBF is not an uncommon occurrence in either method. XEN has a higher rate of PBF of about 22 to 62% [5, 6, 9–11]. Data of the “younger” PF are still scarce, but literature reports at least lower rates than with XEN at 6 to 19% [4, 11–14]. The management of PBF is based on needling or OBR. After XEN, OBR seems to have better SR compared to needling and is applied in many centers as the treatment of choice following PBF [6]. The applicability of these results of OBR as management of PBF after PF is questionable considering the different primary surgical techniques (XEN ab interno, PF ab externo).

In our study, a significant IOP reduction following OBR was achieved in both groups (excluding eyes undergone re-surgeries). After XENR, Steiner et al. reported an IOP reduction of 29.6% at an average of 7.3 months postoperatively (reduction from 22.0 to 15.5mmHg) [6]. Linton et al. reported a 37.5% IOP reduction (26.1 to 16.3mmHg) one year following OBR [15]. These data are comparable to our 6-months-results after XENR with an IOP reduction of 31.4% (24.2±4.7 to 13.5±4.6mmHg). To the best of our knowledge, this is the first study to systematically examine the outcomes of OBR after PF, so that a comparison with the literature was not possible.

Regarding NoM, a significant reduction was reported in the literature following XENR (reduction of 48%–65%) [6, 15]. However, in our study, we did not observe a reduction of NoM 6 months following OBR neither after XENR (0.7±1.3 to 0.4±0.8, *p*=0.641) nor after PFR (1.2±1.3 to 1.0±1.5, *p*=0.362). The indication of OBR seemed to occur earlier in our study population with less attempted IOP reducing local medication in case of PBF. As patients received the first surgery mainly after failing to reach their target pressure under maximal tolerated medical therapy with a high proportion of patients already having multiple intolerances, PBF and increased IOP are most likely to be treated surgically than to re-start glaucoma medication. Consequently, as low NoM was observed preoperatively, a significant reduction in NoM was not achieved. Correspondingly, significantly less NoM was used in both groups before OBR than before primary surgery (*p*<0.001 at XENR and PFR, see Table 1).

Regarding SR, we observed significantly higher rates of CS and QS at 6 months after XENR compared with PFR in this study (58.6% vs. 30.4%, *p*=0.043). At this time, similar rates were observed for both CS and QS within each group. This difference may be attributed to our preference for re-operation in cases of OBR failure rather than restarting IOP-lowering medications. SR after XENR in our study align with those published by Steiner et al. (QS 50.7%) and Linton et al. (QS 56% and CS 44%) after 1 year [6, 15]. Those SR were observed 6 months later than ours, but with less strict criteria for success, using a cut-off of 21 mmHg in contrast to our cut-off of 18 mmHg for postoperative IOP. We are not aware of any other study reporting SR following OBR after PF.

The better SR in XENR may be explained through a switch in the surgical technique from primary surgery as a less traumatic ab interno-implantation to the revision as an ab externo-procedure. In the primary surgery, the implant placement is relatively uncontrolled, leading to uncertainty regarding its subconjunctival, intra- or subtenon position. Conversely in the ab externo-procedure, Tenon’s capsule is additionally separated from the sclera and the implant is positioned in the subtenon space. In addition, the MMC is applied then in a more controlled manner in the now surgically created episcleral space, where its cytostatic effect is more needed. Therefore, the combination of better placement of the XEN and controlled application of MMC may contribute to OBR achieving better SR after XEN.

In PFR, the primary procedure of PF-Implantation and the OBR are comparable as ab externo-procedures. The conjunctiva and tenon have already undergone a significant surgical trauma during the first surgery so that a second (major) trauma seems to correlate with increased scarring and increased rate of failure. The more invasive approach of primary PF implantation is however correlated with lower revision rate compared to XEN, but in the case of a PBF, an OBR may not bring an additional benefit as in the case following XEN. Alternative strategies such as higher dosages or longer application of cytostatic agents or prophylactic use of 5-FU should be considered in order to improve the chances of success. Still, this might correlate with higher rates of complications [16].

Both XENR and PFR showed acceptable safety profile in this study without serious complications as most complications were treated conservatively.

Our study has some limitations, because of the retrospective design and the use of different antimetabolites with different application times during OBR (intraoperative MMC, postoperative 5-FU, or a combination of both). The decision which antimetabolite to use was taken by the surgeon and depended on the clinical judgment. Despite the shorter application time of MMC in the XENR group, we observed better success rates, supporting our main findings. A uniform recommendation regarding the use of MMC in filtering surgery is currently not available. Still, there are indications of better SR with higher MMC concentrations [17, 18].

The need for surgical interventions in case of PBF following XEN or PF is often considered as failure of primary surgery. Our results, however, show that acceptable SR still can be achieved in these cases using OBR, especially following XEN. In the case of PF, failure rate of OBR at 6 months was by 69.6%, which emphasizes the need for a thorough discussion of the benefits and alternatives with patients. Longer application times of MMC and extensive use of postoperative 5-FU may improve these results. Still, since filtering surgery is primarily indicated in cases resistant to medical therapy, the rehabilitation of existing implants respectively their blebs should not be ignored with reasonable SR and low complication rates, especially considering that the alternative often involves more invasive procedures such as glaucoma drainage implants or cyclodestructive procedures.
